# Artificial intelligence: A strategic opportunity for enhancing primary care in South Africa

**DOI:** 10.4102/safp.v64i1.5596

**Published:** 2022-09-01

**Authors:** Ramprakash Kaswa, Arun Nair, Shane Murphy, Klaus B. von Pressentin

**Affiliations:** 1 Department of Family Medicine and Rural Health, Walter Sisulu University, Mthatha, South Africa; 2 Mthatha General Hospital, Mthatha, South Africa; 3 Department of Family Medicine, University of the Free State, Kimberley, South Africa; 4 Robert Magaliso Sobukwe Hospital, Northern Cape Department of Health, Kimberley, South Africa; 5 Mediclinic Pietermaritzburg, Mediclinic Southern Africa, Pietermaritzburg, South Africa; 6 School of Public Health and Family Medicine, Faculty of Health Sciences, University of Cape Town, Cape Town, South Africa

The use of artificial intelligence (AI) is accelerating at an astounding pace in the healthcare industry. Artificial intelligence, a core component of the fourth industrial revolution, is an important nonmedical intervention to support healthcare system responsiveness to society’s emerging health and wellness needs. While AI would enable an intelligent healthcare future, there remain many unanswered questions related to accountability, fairness, privacy, reliability, transparency and safety.^1^ A 2020 scoping review confirmed the potential for AI in strengthening primary care practice and research, especially considering our scope of practice and the exponential growth of information. The authors of this review warned that its usefulness is not guaranteed because of barriers to adopting AI, including concerns about unanticipated outcomes.^2^ In addition, implementing AI in an equitable manner requires our urgent attention to ensure universal access to the potential benefits of AI.

Artificial intelligence is defined as the automation of activities associated with human thinking such as decision-making, problem-solving and learning. Artificial intelligence was first used in medicine in the 1970s and was founded on ‘medical expert’ systems (rule-based algorithms) based on Bayesian statistics and decision theory.^3^ This rule-based methodology led to bias (underfitting of data) which failed to account for population variance.^4^ Consequently, later AI incorporated bioinformatics research (data availability and quality), expanding it to medical artificial intelligence (MAI).^3^ Medical artificial intelligence based on data (frequentist theory) is shown to have higher accuracy and reliability. Interestingly, MAI has been piloted in Africa since the 1980s, and previous South African examples of its use include computerised aid to treat (CATT), based on a cost-and-effectiveness algorithm used in drug prescriptions by nurses, as well as a multinomial logistic classifier-based system applied to human resource planning.^3^

Theoretical physicist Stephen Hawking was credited with saying: ‘Our future is a race between the growing power of our technology and the wisdom with which we use it’. Globally, AI-enabled systems are currently transforming the healthcare sector at an unparalleled rate. The rapid growth of AI is due to increased computing power, growth in the big-data phenomenon and substantial investments in research and expansion of basic AI technologies.^5^ Despite the positive effects of AI in the healthcare industry, it is crucial that South Africa has a comprehensive regulatory framework designed to protect the privacy and security of the data collected and used.^5^ This can be done by developing robust safeguards against unauthorised access and use of data. South Africa does not yet have a policy which focuses solely on the use of AI in health but may draw guidance from the National Digital Health Strategy 2019–2024, which looks more broadly at digital health as an extension of previous e-health developments and recognises an emphasis on digital consumers using a wider range of smart devices, together with other innovative and evolving concepts such as the more widespread use of AI, big data and analytics.^6^ Further guidance may be sought from the recent World Health Organization (WHO) recommendations on the ethics of AI.^7^

Key aspects to enable the growth of MAI in the South African healthcare system, including primary care, revolve around data availability and quality, legal and policy guidance (as highlighted above) and the need to invest responsibly and strategically in the necessary infrastructure and an AI or data-literate workforce to ensure AI adoption and application. Electronic health records are critical to improving the quality and availability of data for MAI. The digitisation of healthcare records represents a significant step in developing machine learning techniques, a subset of AI systems in which computer software applications are trained to recognise and learn from patterns in existing datasets.^7^ However, there exist challenges with data quality as well as language and translation barriers, in which cultural metaphors captured in health records as well as user-generated content will have to be ‘deciphered’.^8^

Primary health care represents the foundation of the health services delivery platform in South Africa and hence should be the focus for next-generation epidemic preparedness.^1^ Dr Tedros Adhanom Ghebreyesus, the WHO director-general, urged countries to achieve fundamental readiness to use AI technologies in a responsible and sustainable way.^1^ The WHO’s Global Digital Health Strategy includes recommendations on using digital health interventions to establish robust and resilient health systems.^9^ The discipline of family medicine as well as the broader health and higher education systems will require partnerships with AI scholars and big data scientists, centred around key activities such as research collaborations and codesigning curricula for 21st-century primary care professionals.^10^ As family physicians and primary care providers, we appreciate the need to embrace complexity^11^ and rethink our approach to the polarity represented by the potential harms vs. benefits of embracing AI. Digital health and AI technologies can make the limited time we have with our patients more meaningful.^10^ It is time for AI and primary care to invest in a synergistic relationship.

## References

[1] Ghebreyesus TA, Swaminathan S. Get ready for AI in pandemic response and healthcare. BMJ Opin [serial online]. 2021 [cited 2022 June 12]. Available from: https://blogs.bmj.com/bmj/2021/10/28/get-ready-for-ai-in-pandemic-response-and-healthcare/

[2] Kueper JK, Terry AL, Zwarenstein M, Lizotte DJ. Artificial intelligence and primary care research: A scoping review. Ann Fam Med. 2020;18(3):250–258. 10.1370/afm.2518 32393561 PMC7213996

[3] Owoyemi A, Owoyemi J, Osiyemi A, Boyd A. Artificial intelligence for healthcare in Africa. Front Digit Health. 2020;2:6. 10.3389/fdgth.2020.00006 34713019 PMC8521850

[4] Rahimi SA, Légaré F, Sharma G, et al. Application of artificial intelligence in community-based primary health care: Systematic scoping review and critical appraisal. J Med Internet Res. 2021;23(9):e29839. 10.2196/29839 34477556 PMC8449300

[5] Naidoo S, Bottomley D, Naidoo M, Donnelly D, Thaldar DW. Artificial intelligence in healthcare: Proposals for policy development in South Africa. S Afr J Bioeth Law. 2022;15(1):11–16. 36061984 10.7196/sajbl.2022.v15i1.797 PMC9439582

[6] National Department of Health. National Digital Health strategy for South Africa 2019–2024 [homepage on the Internet]. Pretoria: The Republic of South Africa; 2022 [cited 2022 Jun 12]. Available from: https://www.knowledgehub.org.za/elibrary/national-digital-health-strategy-south-africa-2019-2024

[7] World Health Organization. Ethics and governance of artificial intelligence for health [homepage on the Internet]. Geneva: World Health Organization; 2021 [cited 2022 Jun 12]. Available from: https://www.who.int/publications/i/item/9789240029200

[8] Yin Z, Sulieman LM, Malin BA. A systematic literature review of machine learning in online personal health data. J Am Med Inform Assoc. 2019;26(6):561–576. 10.1093/jamia/ocz009 30908576 PMC7647332

[9] Global strategy on digital health 2020–2025: WHO guidance [homepage on the Internet]. Geneva: World Health Organization; 2021 [cited 2022 Jun 12]. Available from: https://apps.who.int/iris/bitstream/handle/10665/344249/9789240020924-eng.pdf

[10] Liaw W, Kakadiaris I. Artificial intelligence and family medicine: Better together. Fam Med. 2020;52(1):8–10. 10.22454/FamMed.2020.881454 31914179

[11] Von Pressentin KB. Embracing complexity in primary care. S Afr Fam Pract. 2022;64(1):a5519. 10.4102/safp.v64i1.5519 PMC9082284 35532126

